# Changes in intrathoracic pressure, not arterial pulsations, exert the greatest effect on tracer influx in the spinal cord

**DOI:** 10.1186/s12987-022-00310-6

**Published:** 2022-02-08

**Authors:** Shinuo Liu, Lynne E. Bilston, Neftali Flores Rodriguez, Courtney Wright, Simon McMullan, Robert Lloyd, Marcus A. Stoodley, Sarah J. Hemley

**Affiliations:** 1 Department of Clinical Medicine, Faculty of Medicine, Health and Human Sciences, Macquarie University, Macquarie Park, NSW 2109 Australia; 2 Neuroscience Research Australia, Prince of Wales Clinical School, University of New South Wales, Sydney, NSW 2031 Australia; 3 Sydney Microscopy and Microanalysis, Brain and Mind Centre, The University of Sydney, Camperdown, NSW 2006 Australia; 4 Department of Biological Sciences, Faculty of Medicine, Health and Human Sciences, Macquarie University, Macquarie Park, NSW 2109 Australia

**Keywords:** Cerebrospinal fluid, Spinal cord, Intrathoracic pressure, Hypertension, Tachycardia, Respiration

## Abstract

**Background:**

Cerebrospinal fluid (CSF) circulation in the brain has garnered considerable attention in recent times. In contrast, there have been fewer studies focused on the spine, despite the expected importance of CSF circulation in disorders specific to the spine, including syringomyelia. The driving forces that regulate spinal CSF flow are not well defined and are likely to be different to the brain given the anatomical differences and proximity to the heart and lungs. The aims of this study were to determine the effects of heart rate, blood pressure and respiration on the distribution of CSF tracers in the spinal subarachnoid space, as well as into the spinal cord interstitium.

**Methods:**

In Sprague Dawley rats, physiological parameters were manipulated such that the effects of spontaneous breathing (generating alternating positive and negative intrathoracic pressures), mechanical ventilation (positive intrathoracic pressure only), tachy/bradycardia, as well as hyper/hypotension were separately studied. To investigate spinal CSF hydrodynamics, in vivo near-infrared imaging of intracisternally infused indocyanine green was performed. CSF tracer transport was further characterised with in vivo two-photon intravital imaging. Tracer influx at a microscopic level was quantitatively characterised by ex vivo epifluorescence imaging of fluorescent ovalbumin.

**Results:**

Compared to mechanically ventilated controls, spontaneous breathing animals had significantly greater movement of tracer in the subarachnoid space. There was also greater influx into the spinal cord interstitium. Hypertension and tachycardia had no significant effect on spinal subarachnoid spinal CSF tracer flux and exerted less effect than respiration on tracer influx into the spinal cord.

**Conclusions:**

Intrathoracic pressure changes that occur over the respiratory cycle, particularly decreased intrathoracic pressures generated during inspiration, have a profound effect on tracer movement after injection into spinal CSF and increase cord parenchymal tracer influx. Arterial pulsations likely drive fluid transport from perivascular spaces into the surrounding interstitium, but their overall impact is less than that of the respiratory cycle on net tracer influx.

**Supplementary Information:**

The online version contains supplementary material available at 10.1186/s12987-022-00310-6.

## Background

The interaction between CSF and interstitial fluid (ISF) is vital for many homeostatic functions in the central nervous system (CNS) [1–3]. Many investigators have proposed that the interface between the two fluids occurs at spaces around penetrating blood vessels—the perivascular spaces—with additional interactions via transpial and transependymal routes [3–5]. The precise anatomical configuration of the perivascular space, as well as the mechanisms that mediate fluid and solute exchange, have not been fully elucidated [6]. In the brain, there are compelling data, largely derived from human magnetic resonance imaging (MRI) and computational modelling studies, that physiological forces such as arterial pulsations and respiratory pressures drive fluid flow in the subarachnoid space [7–10]. Negative intrathoracic pressures generated during the inspiratory period of the respiratory cycle produce high CSF flow, overshadowing the cardiac component [11–14]. MRI studies have been critical for recent advances in imaging CSF flow, however, they are limited by their spatial resolution, preventing the study of fluid and solute exchange between CSF and CNS tissue. Currently, only invasive animal experiments can garner this information.

An in vivo two-photon excitation imaging study investigating CSF flow in mouse brain suggested that increasing blood pressure reduced the overall flow in perivascular spaces around arterioles [15]. However, the relative influence of respiration vs arterial pulsations has not been studied in animals. In addition, the dynamics that mediate flow in the spinal cord cannot simply be extrapolated from intracranial studies. Previous work in rat spinal cord by our group, albeit using ex vivo studies, has demonstrated shared pathways for CSF tracer transport around peri-arterial and peri-venous spaces [16], which is in contrast to the glymphatic theory reported in some studies of the mouse brain [17–19]. Not only is the spinal cord parenchyma anatomically different compared to the brain (the grey and white matter are reversed), but the extradural venous anatomy is configured such that there is direct exposure of the epidural venous plexus to intrathoracic pressures [7]. There have been no previous attempts to independently examine the effects of blood pressure, heart rate and negative intrathoracic pressures generated during inspiration on spinal cord fluid dynamics.

To comprehensively assess the impact of cardiorespiratory drivers on spinal CSF flow and CSF/ISF exchange, fluorescent tracers were injected into the cisterna magna of Sprague Dawley rats. To evaluate cervicothoracic spinal CSF bulk flow, a novel in vivo, near infrared (NIR) imaging technique using indocyanine green (ICG) as a CSF tracer was employed. Two-photon excitation imaging was utilized to characterize the movement of individual molecules (1.0 µm FluoSpheres™) in vivo at the microscopic level. Finally, we performed ex vivo analysis of tracer redistribution microscopically in the spinal interstitium (through axial sections of the spinal cord). The effects of *hypertension* (pharmacologically induced), *heart rate* (induced by sinoatrial node pacing) and *negative intrathoracic pressure* in spontaneously breathing rats were compared to control animals maintained using positive pressure ventilation. Transport of fluorescent tracers in the spinal cord were investigated after manipulation of each of the three physiological variables. It was hypothesised that negative intrathoracic pressures would result in greater molecular transport/CSF flow, while elevated pulse pressure and tachycardia would reduce net movement.

## Materials and methods

Male Sprague Dawley rats (aged between 8 and 12 weeks and weighing 280–430 g) were used in all experiments. Ethics approval was obtained from the Animal Ethics Committees of Macquarie University (Protocol No. 2016/032) and the University of Sydney (Protocol No. 2018/1402).

### Surgical preparation

After induction of general anaesthesia with 5% isoflurane in oxygen, each animal was positioned supine on a heating pad and maintained under anaesthesia with isoflurane in 0.2 L/min of oxygen. Heart rate, oxygen saturation, respiratory rate, and temperature were continuously monitored by pulse oximetry and rectal thermometer. Rats undergoing two-photon excitation microscopy were pre-medicated with subcutaneous midazolam (1 mg/kg) and buprenorphine (0.05 mg/kg) prior to anaesthesia with isoflurane.

A right transverse inguinal incision was made, and the femoral neurovascular bundle was exposed for cannulation by arterial and central venous polyethylene catheters. This was followed by a midline suprasternal incision. The trachea was exposed for insertion of an endotracheal tube. This was connected to a respiratory circuit, delivering isoflurane in oxygen. The egress tubing was connected in series to a capnometer (Capstar-100, CWE Inc., Ardmore, PA, USA), for continuous end-tidal carbon dioxide (CO_2_) monitoring, as well as to a custom-made respiratory circuit pressure manometer. At this point, for the subset of rats where heart rate was modulated, a 3-cm length of the right external jugular vein was dissected out. A custom manufactured atrial pacing wire was inserted into this vein to rest just above the sinoatrial node. The pacing wire was connected to an isolated pulse stimulator (A-M Systems Inc, model 2100) [20].

**Fig. 1:**
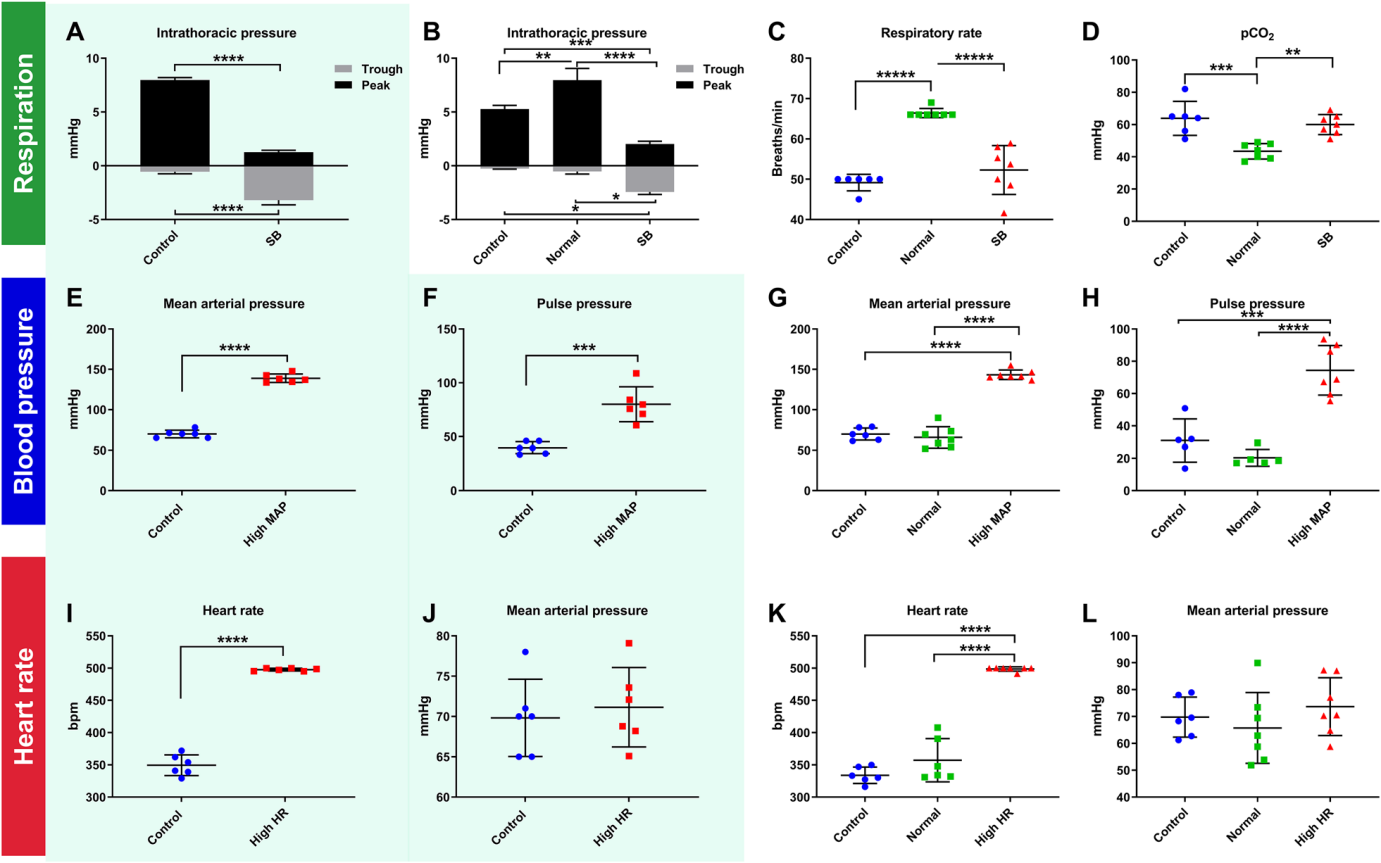
Modulation of respiration, blood pressure and heart rate. The shaded graphs represent data obtained from animals used in the in vivo studies and injected with indocyanine green (ICG) (**A**, **E**, **F**, **I**, **J**). The other graphs (**B**–**D**, **G**, **H**, **K**, **L**) represent the animals from the ex vivo studies using fluorescent ovalbumin (AFO-647) as the CSF tracer. **A**–**D** To test how respiration effects CSF flow, rats were anaesthetised and spontaneously breathing (SB) or were mechanically ventilated to normocarbia (*Normal*) or to hypercapnia (*Control*). The peak circuit pressures of *Normal* and *Control* cohorts were significantly higher than that of SB. Similarly, the trough pressures were more negative in the SB group than either of the positive pressure ventilated groups (*Normal* and *Control*). **C** SB rats had significantly lower respiratory rates (around 50 breaths/min) than the *Normal* animals (set at a respiratory rate of 66 breaths/min) but matched that of the *Control* cohort. **D** The lower, physiological pCO_2_ in the *Normal* group reflected the circuit pressure (used as an indirect measure of intrathoracic pressure) and respiratory rate. **E**–**H** To test the effect of blood pressure on CSF flow, phenylephrine infusions were administered to induce hypertension. **E**, **G** Sustained mean arterial pressure (MAP) and **F**, **H** pulse pressure of approximately 140 mmHg and 80 mmHg respectively over 20 min were achieved. These values were twice that of controls. **I**, **K** To investigate the effects of heart rate on CSF flow, tachycardia was achieved by electrically pacing animals to 500 bpm. These animals had heart rates approximately 50% higher than the mechanically ventilated controls, without any blood pressure differences, demonstrated in **J**, **L**. Two tailed Student’s t-test, **p < 0.01, ***p < 0.001, ****p < 0.0001. All error bars are expressed as ± SD, n = 6/7 rats

The animal was then repositioned prone and the respiratory circuit manometer and arterial line were connected to pressure transducers, enabling the continuous measurement of blood pressure and circuit pressure. All vital statistics were recorded continuously for the remainder of the experiment on a data acquisition interface, Power1401 (Cambridge Electronic Device, Cambridge, UK). Arterial blood gas was analysed for pH, partial pressure of oxygen, and partial pressure of CO_2_. Each physiological variable—respiration, blood pressure and heart rate—was then manipulated in isolation to investigate its effect on spinal subarachnoid space flow and parenchymal inflow (Fig. 1).

### Modulation of physiological parameters

#### Respiration

To examine the effects of negative intrathoracic pressure generated by spontaneous respiration, rats were either allowed to breathe spontaneously on the respiratory circuit, or a neuromuscular blockade was administered (pancuronium bromide 0.8 mg induction, 0.4 mg/h maintenance) followed by mechanical ventilation using a small animal ventilator (Harvard 7025 Rodent Ventilator, set at a tidal volume of approximately 1.2 mL). Any prolonged vagolytic effects of pancuronium resulting in hypertension and tachycardia were reversed with small boluses of metoprolol (15 mg/kg). The end tidal CO_2_ was adjusted to a physiological range of 3.5–4.5%. In anaesthetised spontaneously breathing rats, bradypnoea of 50–55 breaths/min was observed, with resultant CO_2_ retention and respiratory acidosis. Therefore, control animals underwent positive pressure ventilation with a respiratory rate and blood gas profile matching that of their spontaneous breathing counterparts. Since these animals were under isoflurane anaesthesia they had low heart rates and blood pressures. This control group was also used for the blood pressure and heart rate experiments. Other variables including mass, heart rate, and mean arterial pressure (MAP) were kept constant between spontaneous breathing and control groups.

#### Blood pressure

To examine the effects of blood pressure, hypertensive rats were compared to the mechanically ventilated group(s), which had an approximate MAP of 70 mmHg. In hypertensive rats, the MAP was raised to a target of 140 mmHg (a ~ 40% increase from the normal base value, or approximately double that of controls) by an escalating infusion of phenylephrine (20–200 µg/kg/min), a selective α_1_-adrenergic receptor agonist. In order to maintain a constant heart rate and prevent baroreflex compensation, the nicotinic receptor antagonist hexamethonium (20–25 mg/kg) was administered. All hypertensive animals were mechanically ventilated to relative hypercapnic levels (matching the *Control* group). Mass, heart rate, and ventilation were kept constant between hypertensive and control groups.

#### Heart rate

To examine the effect of heart rate, tachycardic rats were compared to the mechanically ventilated group(s), which had an approximate heart rate of 330 beats/min (bpm). A pacing wire (described above) was connected to an isolated pulse generator (A-M Systems Inc, model 2100). A square pulse duration of 2 ms and an amplitude aiming for 1.0 V was used to generate a heart rate of at least 500 bpm. There was no appreciable change in blood pressure on pacing, even over prolonged periods. All tachycardic animals were mechanically ventilated to a relative hypercapnic level (matching the *Control* group). Mass, MAP, and ventilation were kept constant between tachycardic and control groups.

#### Ventilation rate/blood gas profile

To determine if blood pH, partial pressure of CO_2_, and rate of ventilation have an effect on ISF/CSF fluid dynamics, an additional control group (*Normal*), maintained with positive pressure ventilation to a higher respiratory rate of 66 breaths/min, which normalised partial pressure of CO_2_ and pH, was introduced. Since the likely consequence of acidosis is dilatation of penetrating arterioles [21], it was hypothesised that the greatest effect of altering respiratory rate would be observed on tracer influx into the spinal cord parenchyma. Thus, a higher respiratory rate control group with normal physiology was introduced in the ex vivo studies investigating the effect of respiration, blood pressure, and heart rate on the distribution of tracer.

### Surgical procedures for investigation of fluid dynamics

After the desired physiological parameter target was reached and maintained, one of three surgical procedures was performed to investigate fluid transport in the spinal subarachnoid space, and into the spinal cord. CSF tracer movement was assessed using one of the following fluorescent tracers injected into the cisterna magna: ICG (Verdye, Aschheim-Dornach, Germany); 1 μm FluoSpheres™ (13% v/v: ThermoFisher Scientific, Massachusetts, USA), Ovalbumin Alexa-Fluor®-647 conjugate (AFO-647: Life Technologies, Victoria, Australia). CSF tracer movement was imaged using NIR intravital imaging, two-photon microscopy, and ex vivo microscopy.

#### NIR intravital imaging

To characterise the movement of CSF in the cervicothoracic subarachnoid space in vivo, NIR imaging of an intracisternally injected fluorescent CSF tracer, ICG, was performed. Under an OPMI Pentero 800 microscope (Carl Zeiss, Oberkochen, Germany) a midline dorsal incision was made, followed by muscle dissection to expose the atlanto-occipital membrane and the dorsal bony elements from C1 to T2 (these were kept intact). The cisterna magna was accessed via a single pass through the atlanto-occipital membrane of a 30G Hamilton needle loaded onto a glass syringe (Hamilton Company, Nevada, USA) mounted on a stereotactic frame. Approximately 1 mm of the needle tip was left in situ, and cyanoacrylate glue was applied around the cannulation site to prevent CSF leakage. After the physiological targets were achieved, the Pentero NIR camera function, IR800, was then activated (set at a zoom of 4.0× and a focal length of 300 mm) to encompass the surgical field from the craniocervical junction to T2. A 10 μL aliquot of 5 mg/mL ICG (Verdye, Aschheim-Dornach, Germany) was delivered into the cisterna magna at 33 nL/s via an Ultramicro pump (World Precision Instruments, Florida, USA). The intraoperative in vivo fluorescence of redistributed tracer and the corresponding operative field under white light were continuously and simultaneously recorded from the start of injection for 20 min. The animal then underwent transcardiac perfusion with heparinised 0.1 M phosphate buffered saline (PBS) followed by 4% paraformaldehyde (PFA) (Lancaster Synthesis, Pelham, New Hampshire).

#### In vivo two-photon excitation microscopy

To study the dynamic interaction between CSF and the spinal cord in the subarachnoid space and paravascular space, multiphoton intravital microscopy was employed to evaluate the real-time flux of fluorescent microspheres. After extensive muscle dissection of the upper thoracic levels, T3 and T4 laminectomies were performed. A polyethylene sheath attached to a rubber ring was used to create a custom-made tubular spinal imaging window. A rapid-setting silicon glue (Twinsil®, Picodent, Wipperfürth, Germany) was used to adhere the window to the surrounding soft tissue, creating a watertight seal for the immersion fluid. The cisterna magna catheter was fashioned from the 3 mm tip of a 29G needle fitted into a polyvinyl chloride catheter. The catheter was prefilled with CSF tracer mixture and loaded onto a 10 μL Hamilton needle and syringe, inserted into the cisterna magna and cyanoacrylate glue applied to secure the cannula in place. The instrumented animal was moved *en masse* to the stage of the Leica SP8 Deep In Vivo Explorer (DIVE) multi-photon microscope (Leica Microsystems, Wetzlar, Germany), equipped with non-descanned spectral tunable detection, an InSight™X3 Ti-Sapphire (Spectra-Physics, MKS Instruments, Inc. USA) laser system tunable up to 1300 nm (maintained at constant power at a range of 10–20%) and a Vario Beam Expander (VBE) that allows tuning for best depth penetration and for best resolution. There was continuous physiological recording throughout the experiment. A target leptomeningeal vessel measuring 150–200 μm in width was acquired, and a 100–200 μL bolus of 2 mg/mL fluorescein dextran or tetramethylrhodamine dextran vascular tracer (2,000,000 MW, ThermoFisher Scientific, Massachusetts, USA) was administered intravenously to delineate the vasculature. In animals that were imaged over an extended time, both tracers were administered, one at the initial imaging time, the second, infused once the initial tracer was no longer clearly visible.

Following manipulation of the target physiological parameter, a 10 μL volume of a CSF tracer mixture of AFO-647 (9 µL of 20 µg/µL solution) and 1 μm blue-green fluorescent microspheres (FluoSpheres™) (1 µL of 13% v/v solution), was delivered at 33 nL/s through the cisterna magna catheter. Vascular tracers fluorescein and tetramethylrhodamine, AFO-647 and the fluorescent microspheres were simultaneously excited with a wavelength of 850 nm, emission wavelengths ranges were set at 500–550 nm, 570–620 nm, 660–710 nm and 420–470 nm respectively (detector gains were used between 10.0 and 100.0%). Images were acquired with a × 25 water immersion objective (IRAPO L 25x/1.0W motCORR, Leica Microsystems, Germany). Images and videos were obtained in the *x*, *y* axes. At the completion of imaging, the rats were euthanised by administration of pentobarbital.

### Ex vivo microscopy

The redistribution of fluorescent tracer from the cisterna magna to the cervicothoracic spinal cord interstitium was assessed qualitatively and quantitatively. As described above, a 30G needle was used to inject a 10 μL volume of 20 µg/µL AFO-647 tracer into the cisterna magna at a rate of 33 nL/s. The needle was left in situ ensuring CSF leakage did not occur. After 10 min the animal underwent transcardiac perfusion.

The brain and spinal cord of all animals, except those that had undergone intravital microscopy, were harvested *en bloc*. The spinal cord was then segmented from C2 to T4 after post-fixation and cryoprotection. Each segment was snap frozen, and 40 μm axial sections were acquired and mounted onto glass slides for immunohistochemistry. To label the endothelium, slides were incubated with the primary Rat Endothelial Cell Antibody (RECA-1, Abcam, Cambridge, United Kingdom) in 4% Normal Donkey Serum (NDS), followed by the secondary antibody, anti-mouse IgG Alexa-Fluor® 488 (Molecular Probes, Life Technologies, New York, USA). Smooth muscle cells were then labelled by anti-actin α-smooth muscle-Cy3™ antibody (SMA, Sigma-Aldrich, St. Louis, Montana). Immunohistochemistry was not performed on specimens exposed to ICG as the processing degraded the tracer.

All spinal cord axial sections from C2 to T4 were imaged with a Zeiss Axio Imager fluorescence microscope (Carl Zeiss Microimaging GmbH, Germany). In axial sections that had undergone immunohistochemistry, arterioles were positive for RECA-1 and SMA, whereas venules and capillaries were labelled by RECA-1 only (vessels that had a luminal diameter < 6.5 μm were classified as capillaries). Further delineation of vascular structures and the central canal was undertaken with confocal microscopy (LSM 880, Carl Zeiss Microimaging GmbH, Germany).

### Image processing and analysis

#### CSF tracer transport in the subarachnoid space

In the NIR study, videos of the intraoperative fluorescence as well as the corresponding white light channel were converted to image stacks using Image J, version 1.46r [22]. The background signal was subtracted in each frame to give the mean pixel intensity (a measure of fluorescence intensity) at each spinal level from C2 to T2. Fluorescence intensity was measured every second for 20 min. Pulse-like wavefronts of tracer fluorescence were observed to propagate rostrocaudally from the point of injection and were particularly prominent between C1 and C2. Surface plots of fluorescence intensity versus displacement were constructed in ImageJ to reconstruct this phenomenon. The peak amplitude of these tracer wavefronts was tracked (via the surface plots) between C1 and C2 to compute its velocity in pixels/s.

The time-series videos obtained from the two-photon excitation microscopy had a bit depth of 16-bit, each with spatial dimensions of 256 × 256 pixels and temporal resolution of 24 frames per sec. The blue channel captured the fluorescent microspheres. The red and green channels captured the tetramethylrhodamine-dextran and fluorescein-dextran, respectively, in the vasculature. The dextrans were injected at different times, so in each video, the brightest dextran was kept and the other channel, either the red or green channel, was deleted. Images were processed using a Gaussian Filter, Median Filter and background subtraction in Imaris × 64 v9.0.2 (Bitplane, http://www.bitplane.com/).

The videos were then analysed using the TrackMate plugin in FIJI (NIH, https://imagej.nih.gov/ij/). The trajectory of each microsphere was manually tracked. Only microspheres that could be tracked over multiple frames with confidence were included. Static microspheres were excluded. Quantification of sphere velocity and displacement were recorded. However, since we could only track a small number of spheres in a limited number of animals/regions of interest this analysis could not be validated. Instead, qualitative observations were made based on the videos generated displaying the tracked microspheres for each animal.

#### Ex vivo experiments on tracer influx into the spinal cord

In fluorescence microscopic axial sections, the integrated density of the CSF tracer (mean pixel density multiplied by area) was calculated. The integrated density of the whole spinal cord, the white matter, and grey matter were separately calculated in each axial section. The dura and nerve roots were meticulously excluded. At least three sections were analysed per level from C2 to T4, and averaged. The white and grey matter have different densities of arterioles and the extracellular space in each of these areas is configured differently [16]. We hypothesise that perivascular spaces are important routes of fluid and solute exchange. This means that hypertension/tachycardia might exert different effects on fluid inflow in grey matter because there are more arterioles there. The white matter, on the other hand, is directly exposed to the subarachnoid space which means transpial movement is likely to be more evident here than in the grey matter.

In ex vivo tracer experiments, the number of fluorescent deposits of tracer larger than 50 pixels within the grey matter were counted. These aggregations were too large to indicate tracer diffusion and represented tracer accumulating around blood vessels. These were referred to as “grey matter vascular events”. The number of discrete “events” was assumed to reflect accumulation of tracer around the perivascular space of grey matter central arteriolar branches. The aggregate area of these “events” was also expressed as a percentage of the total grey matter area.

### Experimental design and statistical analyses

Physiological vital statistics were compared with two tailed Student’s t-test and values expressed as mean ± standard deviation (SD). Where there were three groups, one-way analysis of variance (ANOVA) was employed and adjusted for multiple comparisons with Tukey post-hoc tests. Fluorescence intensities (integrated densities and mean pixel intensities) were compared using two-way ANOVA and adjusted for multiple comparison using Bonferroni’s post-hoc tests. A p value < 0.05 was considered statistically significant in all analyses. All fluorescence values were expressed as mean ± standard error of the mean (SEM). GraphPad Prism (v7.02, GraphPad Software Inc, California) was used to perform all statistical analysis. Graphs were plotted using GraphPad Prism and MATLAB.

## Results

### Respiration is a major driver of CSF tracer transport in the subarachnoid space

**Fig. 2:**
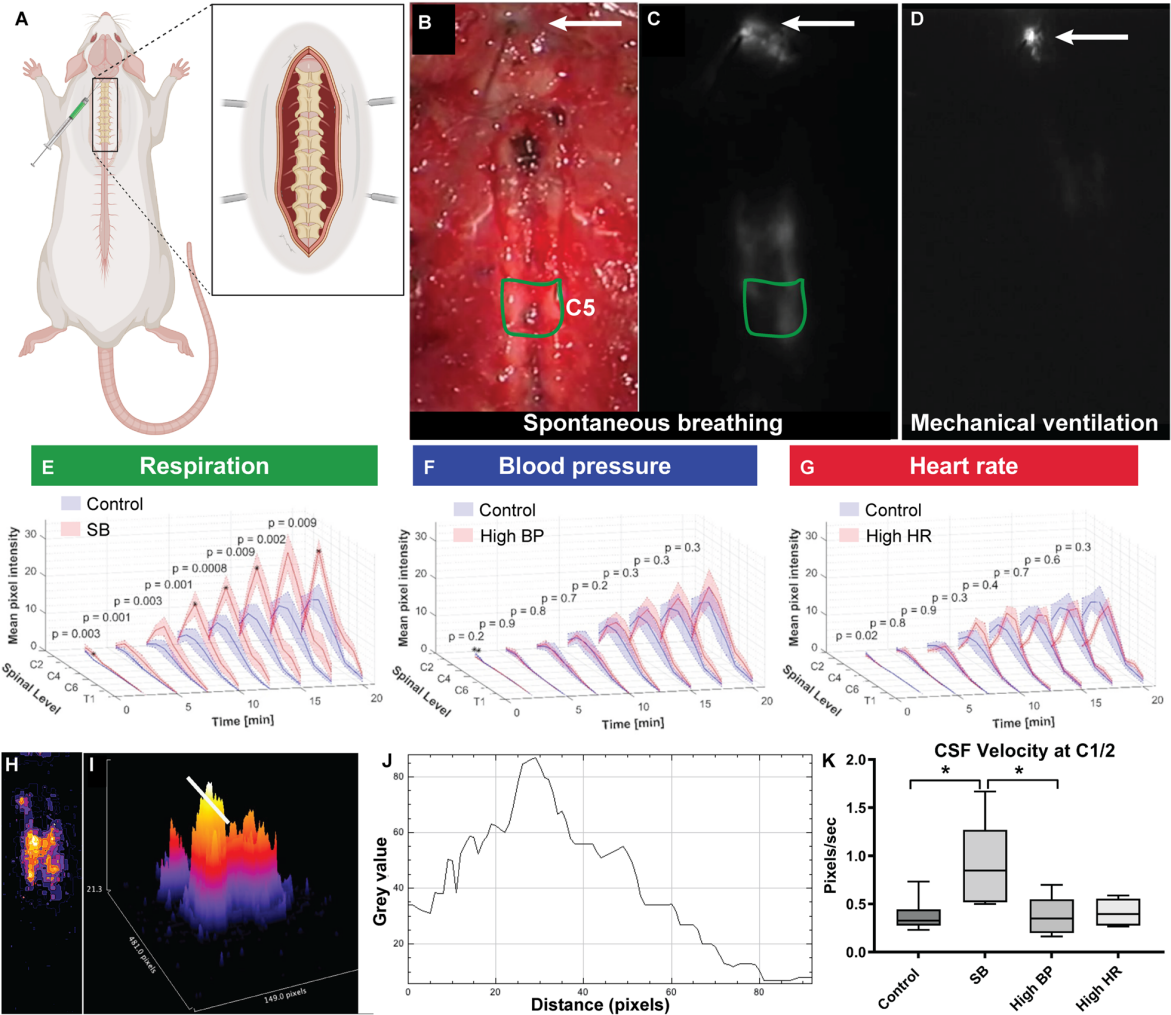
Respiration drives CSF flow in the subarachnoid space. Spinal CSF flow was imaged in live rats using a near infrared (NIR) filter on a commercial operating microscope. **A** Following extensive muscle dissection to expose the bony anatomy from occiput to T2, the CSF tracer indocyanine green (ICG) was infused into the cisterna magna. **B** The intraoperative view under white light was captured and used to identify the injection site (arrow) and each spinal level (an example of a region of interest is shown in green, delineating C5). **C**, **D** The corresponding fluorescent video captured using the NIR filter (Additional file 1: Video S1 and Additional file 2: Video S2) was then used to evaluate fluorescent signal intensity from C2 to T2. Data were collected in real-time over 20 min and measured at 150 s intervals. A representative image from a spontaneous breathing (**C**) and mechanically ventilated (**D**) animal are shown. **E**–**G** The fluorescence intensity (mean pixel intensity) over the 20 min time-point is shown. **E** Pooled data compare the effect of positive pressure mechanical ventilation (*Control*) to spontaneous breathing (SB). Significantly higher fluorescence intensities were measured in SB rats compared with the controls. **F** Compares hypertensive rats (high MAP) with controls (with low MAP). No statistically significant differences were detected at any time up to 20 min. **G** Compares tachycardic rats (high HR) with controls (with low HR). Tracer signal intensities between control and tachycardic rats were similar at all time points except at 2.5 min. Two-way analysis of variance (ANOVA) post hoc Bonferroni’s. All error bars are expressed as ± SEM, n = 6 rats. **H** Heat maps were generated from fluorescence images. In the heat maps, high tracer signal lies towards the yellow, while low signal lies towards the violet end of the spectrum. **I** The corresponding 3D heat map. The white bar marks the interval between C1 and C2. **J** A surface plot of tracer signal intensity demonstrates a pulse like wavefront of CSF tracer. **K** Tracer waves propagated in a caudal direction and their velocities were computed between C1 and C2. In this study, velocity was significantly higher in SB rats compared to controls (*p = 0.04) as well as hypertensive rats (SB vs. BP *p = 0.04). Two-way analysis of variance (ANOVA) post hoc Bonferroni’s. All error bars are expressed as ± SEM, n = 6 rats

Following intracisternal injection of ICG, the distribution of this fluid tracer was qualitatively and quantitatively assessed in real-time in vivo using a NIR camera (Fig. 2, Additional file 1: Video S1, Additional file 2: Video S2). In all rats, ICG tracer migrated caudally along the lateral aspects of the vertebral canal. Over time, increasing fluorescence was detected at the more caudal spinal levels, and the area of highest intensity shifted caudally.

Fluorescence intensities at each spinal level were quantitatively sampled at 150 s intervals after injection (Fig. 2). At every time point analysed after injection of tracer, significantly higher fluorescence intensities were measured in spontaneous breathing rats compared with *Control* animals on positive pressure ventilation (Fig. 2E). Significant difference at C3 was detected 2.5 min after tracer injection (p = 0.003). When hypertensive rats were compared to controls, no statistically significant difference in fluorescence intensity was detected during the 20 min period after intracisternal ICG injection (Fig. 2F). Similarly, the only difference in fluorescence signal intensities between *Control* and tachycardic rats was observed at 2.5 min (Fig. 2G).

A pulse-like wavefront of tracer fluorescence was detected and quantified in the upper cervical levels. The velocity of this wavefront was significantly higher in spontaneous breathing rats compared with the *Control* group (Fig. 2). In spontaneously breathing and positive pressure *Control*, the mean values were 0.9 and 0.4 pixels/s (95% CI 0.04–1.0, p = 0.04 two-tailed t-test), respectively. No significant difference was observed between *Control* and hypertensive or tachycardic cohorts. In these NIR experiments, we have demonstrated that spontaneous respiration, but not arterial pulsations, is associated with greater displacement of injected CSF tracer.

**Fig. 3:**
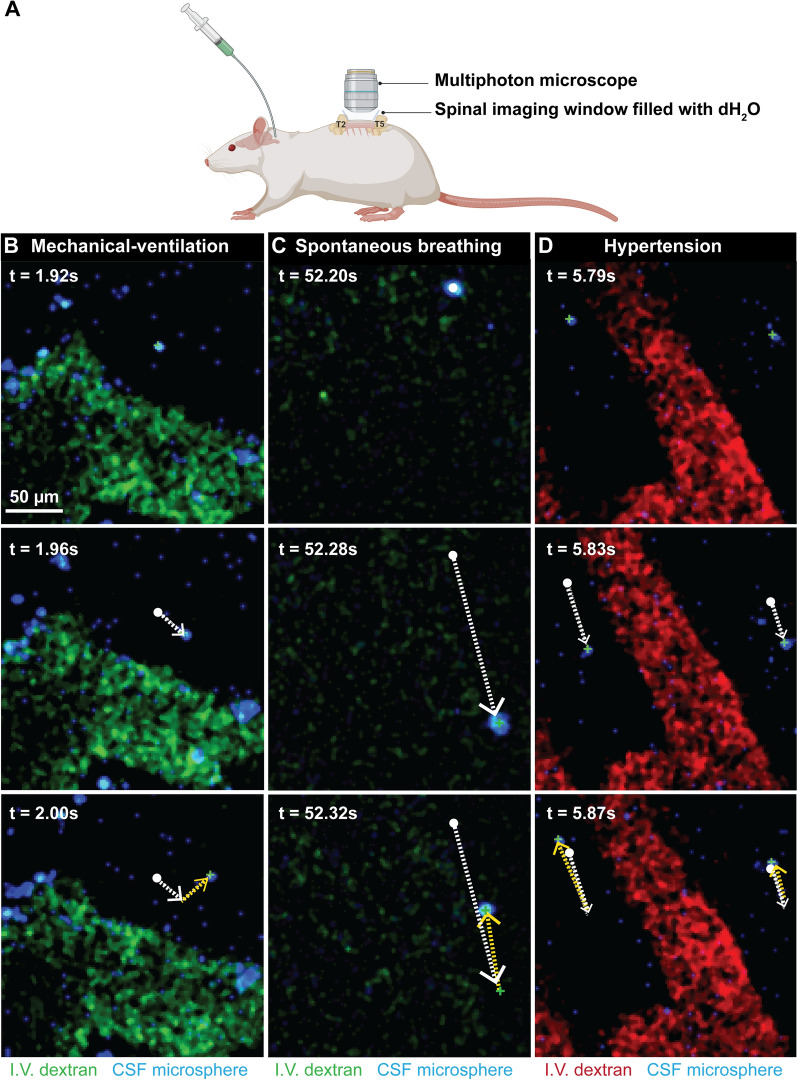
Multi-photon intravital imaging was used to image the movement of intracisternally injected microspheres. Fluorescent dextrans (fluorescein or tetramethylrhodamine) were injected intravascularly to visualise blood vessels. Time-series from positive pressure ventilated controls, spontaneous breathing and hypertensive animals (n = 1–3 animals per group) are shown. **B**–**D** Individual microspheres (blue) were manually tracked and observations noted. Tracked microspheres are labelled with green (+), white solid dots show the start of the track. The white broken arrows show the movement between frames 1 and 2, the yellow broken arrows show the movement between frames 2 and 3. Spontaneous respiration appeared to result in greater displacement of microspheres, likely a consequence of movement in the *z* axis, generating greater movement artefact compared with control animals. With hypertension the particles oscillated about stationary points with minimal net displacement along the length of the blood vessel (Additional file 3: Video S3, Additional file 4: Video S4, Additional file 5: Video S5, Additional file 6: Video S6)

To further determine the effects of each of these altered physiological parameters on fluid exchange at the microscopic level, intracisternally injected fluorescent particles were tracked in real time in vivo at the junction of the subarachnoid and/or perivascular space. A spinal two-photon intravital imaging technique was developed to characterise the motion of injected microspheres, in relation to pial blood vessels, between T2 and T5. Particle movement was dynamic, with displacement of the 1 µm microspheres appearing comparatively greater in spontaneous breathing animals compared to controls (Additional file 3: Video S3, Additional file 4: Video S4), which may account for the higher pulse wave velocities noted above. Hypertension resulted in greater back and forth movement, but it did not appear that this corresponded to an increase in overall displacement of particles (Additional file 5: Video S5). This may account for the relatively reduced CSF velocities calculated by NIR imaging. In controls, spontaneous breathing, hypertensive, and tachycardic animals, microspheres moved in an oscillatory pattern (Fig. 3). Interestingly, there was no evidence microspheres were compelled to oscillate within the paravascular spaces as noted by others in the brain [15]. This may be due to the relatively large particle sizes or that this phenomenon only occurs deeper in the interstitium.

Therefore, spontaneous respiration was associated with greater cervicothoracic CSF tracer movement than positive pressure mechanical ventilation. Increasing the MAP or heart rate resulted in little effect on subarachnoid space molecular transport. The emerging in vivo data suggests greater displacement and mixing may be responsible for this finding.

### Increased ventilation rate and normal physiology enhances tracer influx into the spinal cord

In anaesthetised, spontaneously breathing rats, bradypnea of 50–55 breaths/min was observed, with resultant CO_2_ retention and respiratory acidosis (pCO_2_ 60 ± 6.8 mmHg, pH 7.3 ± 0.05). Hypercarbia is widely believed to induce vasodilatory effects on arteries in the CNS [23]. To eliminate acidosis as a potential confounding variable in the current study we compared our *Control* animals ventilated at a low respiratory rate, resulting in respiratory acidosis (pCO_2_ 63.8 ± 10.6 mmHg, pH 7.3 ± 0.04), and animals ventilated at a respiratory rate of 66 breaths/min (*Normal*), which normalized the partial pressure of CO_2_ and pH (43.4 ± 4.8, 7.4 ± 0.06, respectively). These two controls were used to assess the effects, if any, of blood pH, partial pressure of CO_2_ and rate of ventilation on ISF/CSF fluid dynamics. As arteriolar vasodilation was thought to exert the greatest effect on fluid transport at the level of the perivascular space, this *Normal* cohort, was established only in ex vivo experiments involving intracisternal injection of AFO-647.

In axial microscopic sections, there was an overall higher fluorescence intensity in the cohort ventilated at a higher rate compared with the *Control* group (p = 0.0008). There was also higher AFO-647 signal intensity within the white matter (p = 0.0006). On post hoc analysis, these differences were significant only at C2 and C3 for whole spinal axial section (p = 0.003 and 0.03, respectively) and white matter (p < 0.0001 and 0.0005, respectively). No differences were detected between the two groups for grey matter fluorescence or “perivascular events”.

### Negative intrathoracic pressure promotes tracer influx into white and grey matter

Our in vivo studies showed that exposure to negative intrathoracic pressures during spontaneous breathing facilitated the dispersion of tracers within the spinal CSF compartment but did not indicate whether such effects influenced ISF dynamics. A previous study by our group indicated that tracer injected into the spinal grey and white matter had distinct patterns of distribution. Tracer injected into the grey matter was transported radially, while transport occurred along the axonal fibres in the white matter [16]. Given this, we investigated CSF tracer in the whole spinal cord and separately in the grey and white matter.

**Fig. 4:**
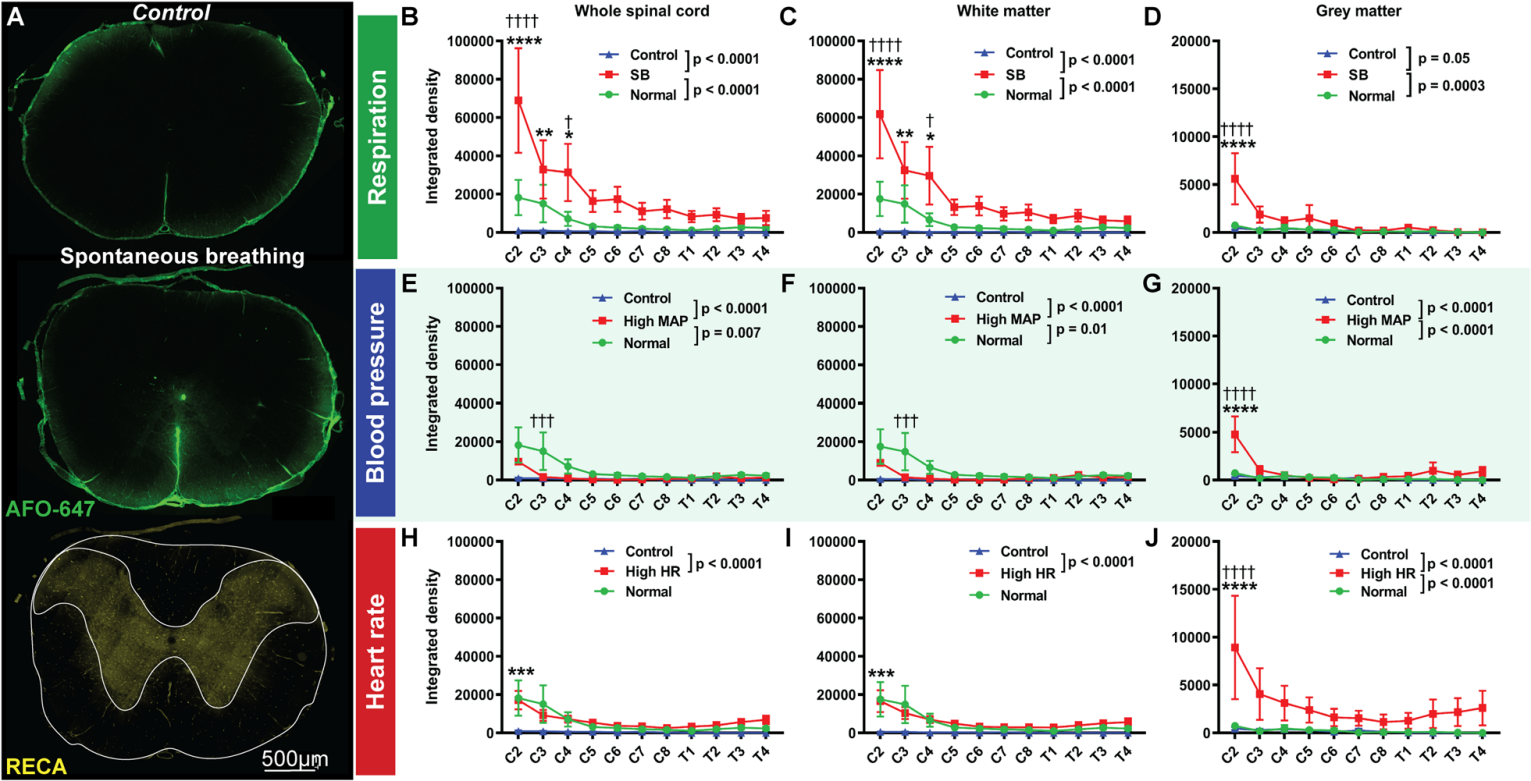
Intrathoracic pressure promotes fluid flow into the spinal white and grey matter. **A** Quantitative analysis of fluorescent ovalbumin (AFO-647) inflow into the spinal interstitium after intracisternal infusion was assessed on microscopic images. Grey and white matter were delineated on the channel immunostained with rat endothelial cell antigen (RECA). Microscopic fluorescence intensities within the spinal cord from: **B**, **E**, **H** whole coronal sections; **C**, **F**, **I** white matter; and **D**, **G**, **J** grey matter were measured at every level from C2 to T4. Experiments investigating the effects of: **B**–**D** respiration; **E**–**G** blood pressure; and, **H**–**J** heart rate on fluid flow were carried out. **B**–**D** There was significantly higher fluorescence within the whole axial section (**B**), grey matter (**D**), and white matter (**C**), in spontaneous breathing (SB) rats compared with the control groups. On post hoc analysis, the difference between SB and controls reached significance at C2 in all compartments of the spinal cord, from C2 to C4 compared to *Controls*, C2 and C4 compared to *Normal* controls in the white matter, and at C2 only in the grey matter. In the white matter and whole axial section (**E**–**G**), the intensity of AFO-647 signal was significantly lower in hypertensive rats compared with controls ventilated at a higher respiratory rate and normalised partial pressure of CO_2_ and pH (*Normal*). However, compared with the matched *Controls*, tracer fluorescence was higher in hypertensive rats. In the grey matter (**G**), tracer signal intensity was higher in hypertensive rats than either of the control groups. **H**–**J** Within the whole axial section and white matter, there was no difference in AFO-647 intensity between tachycardic rats and *Normal* control, but the tachycardic cohort had significantly higher fluorescence than *Controls*. Tracer signal was significantly higher in the grey matter in tachycardic animals compared with controls (**J**). Two-way analysis of variance (ANOVA) post hoc Bonferroni’s, *p < 0.05, **p < 0.01, ***p < 0.001, ****p < 0.0001, mean ± SEM, n = 6/7 rats. (*) denotes significant difference to *Controls*, (†) denotes significant difference to *Normal* controls

**Fig. 5:**
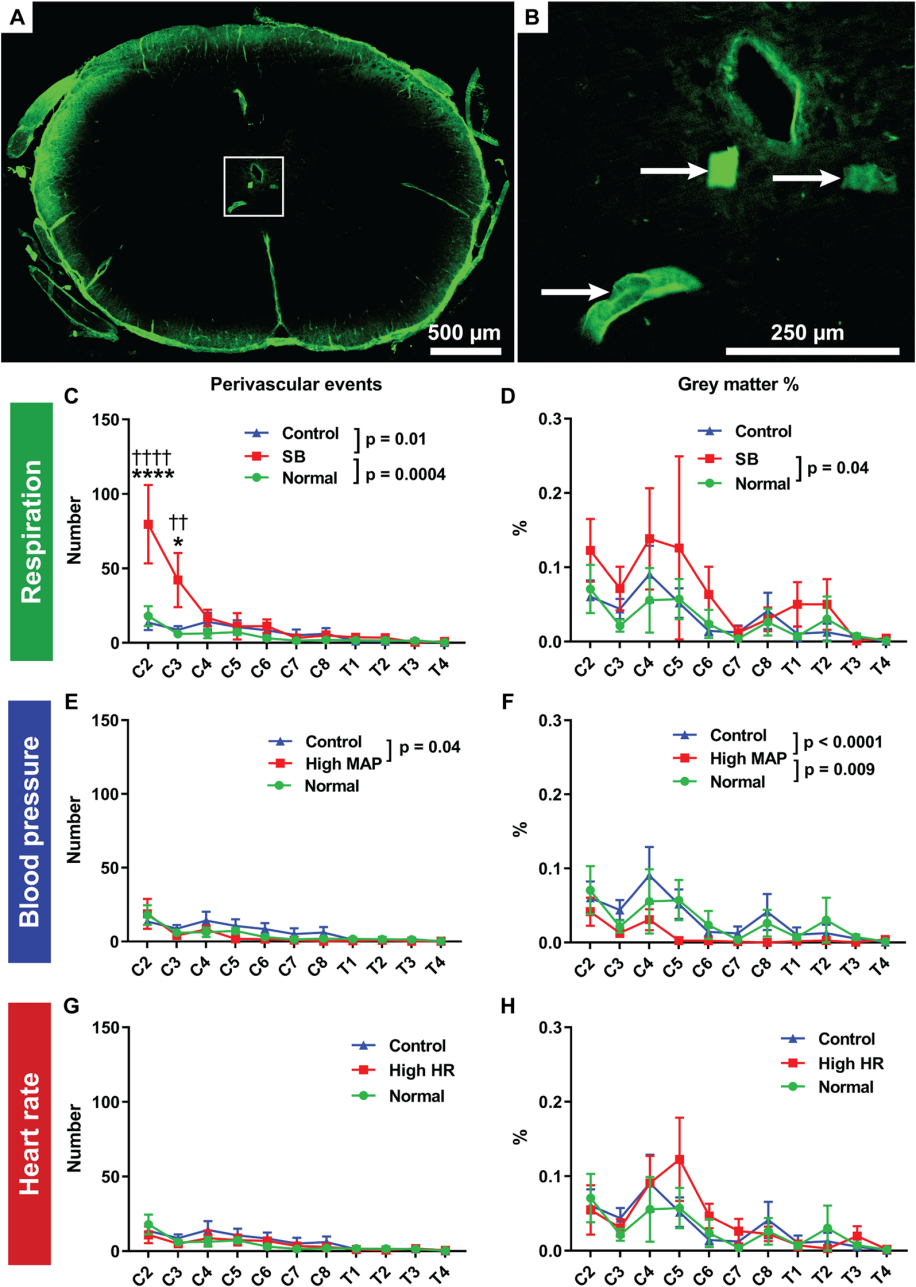
Adjunctive analysis of spinal interstitial inflow. **A**, **B** The number of fluorescent “perivascular events” within the grey matter (arrows) was assumed to represent deposits of fluorescent ovalbumin (AFO-647) around perivascular spaces of central arteriolar, venular or capillary branches, a measure of central cord inflow. **C** In spontaneous breathing (SB) rats, there were greater number of “events” compared with control groups, with C2 and C3 reaching significance on post hoc analysis. **E** More “events” were found in the Low MAP *Control* group than in hypertensive rats. **G** There was no difference between the tachycardic animals and either control groups. **D**, **F**, **H** A measure of central spinal cord inflow was “grey matter %” (the aggregate area of “perivascular events” as a percentage of the total grey matter area). **D** SB was the only variable that resulted in significantly greater “grey matter %” compared with controls. **F** There was a significant reduction in “grey matter %” in hypertensive animals. **H** Tachycardia resulted in no difference compared with controls. Two-way analysis of variance (ANOVA) post hoc Bonferroni’s, (*) denotes significance to *Control* animals, (†) denotes significance to *Normal* controls, *p < 0.05, **p < 0.01, ****p < 0.0001. All error bars are expressed as ± SEM, n = 6/7 rats

Influx of AFO-647 tracer into the spinal parenchyma was measured in axial cross-sections from spinal levels C2–T4. There was significantly higher tracer signal within the whole section of the spinal cord, as well as within constituent grey and white matter compartments, in spontaneous breathing rats compared with the positive pressure ventilated controls (p < 0.0001 for both controls) (Fig. 4B–D, refer to figure for p values). There was significantly greater number of fluorescent deposits (representing tracer associated with blood vessels or “perivascular events”) in the grey matter of spontaneous breathing rats compared with controls (*Normal* p = 0.0004, *Control* p = 0.01) (Fig. 5C). Moreover, the proportion of the grey matter area occupied by fluorescence was higher in spontaneous breathing rats (p = 0.04) compared with *Normal* controls (Fig. 5D). On post hoc analysis, more “perivascular events” were found at C2 and C3 in the spontaneous breathing group compared with controls. Thus, differences in AFO-647 transport were the most pronounced at the upper cervical levels.

The overall AFO-647 fluorescence was significantly higher in hypertensive rats compared with the *Control* group but lower compared with *Normal* controls. This was observed within the whole cord and the white matter (Fig. 4E, F). Curiously, within the grey matter there was significantly higher AFO-647 signal in the hypertensive group compared with either controls. This trend reached significance at C2 on post hoc analysis (Fig. 4G). These findings were not borne out in adjunctive analyses (Fig. 5). Compared with hypertensive rats, there were significantly more “perivascular events” (p = 0.04) in the *Control group* and greater percentage of grey matter occupied by tracer (p = 0.0001) in both control cohorts (Fig. 5E, F).

There was higher tracer signal in tachycardic rats compared to the matched *Control* animals (p < 0.0001), but not the *Normal* controls (Fig. 4H, I). Within the grey matter, the overall AFO-647 intensity was significantly higher in tachycardic rats than either control groups (p < 0.0001) (Fig. 4J). Adjunctive analysis of “perivascular events”, and the percentage of grey matter occupied by tracer did not reveal any differences between tachycardic and the control groups (Fig. 5G, H).

Multiple tracer experiments have, thus far, consistently demonstrated that spontaneous breathing enhances CSF tracer deposition in the spinal subarachnoid space as well as transport into the interstitium. The findings from tachycardic and hypertensive rats have been less certain with increasing blood pressure and heart rate driving more CSF tracer into the spinal cord than the matched controls. However, this is offset by increasing respiratory rate and return to normocapnia.

### Fluid influx occurs via transpial and perivascular pathways

Further insights into spinal fluid transport may be gained from qualitative assessment of AFO-647 influx into the interstitium.

**Fig. 6:**
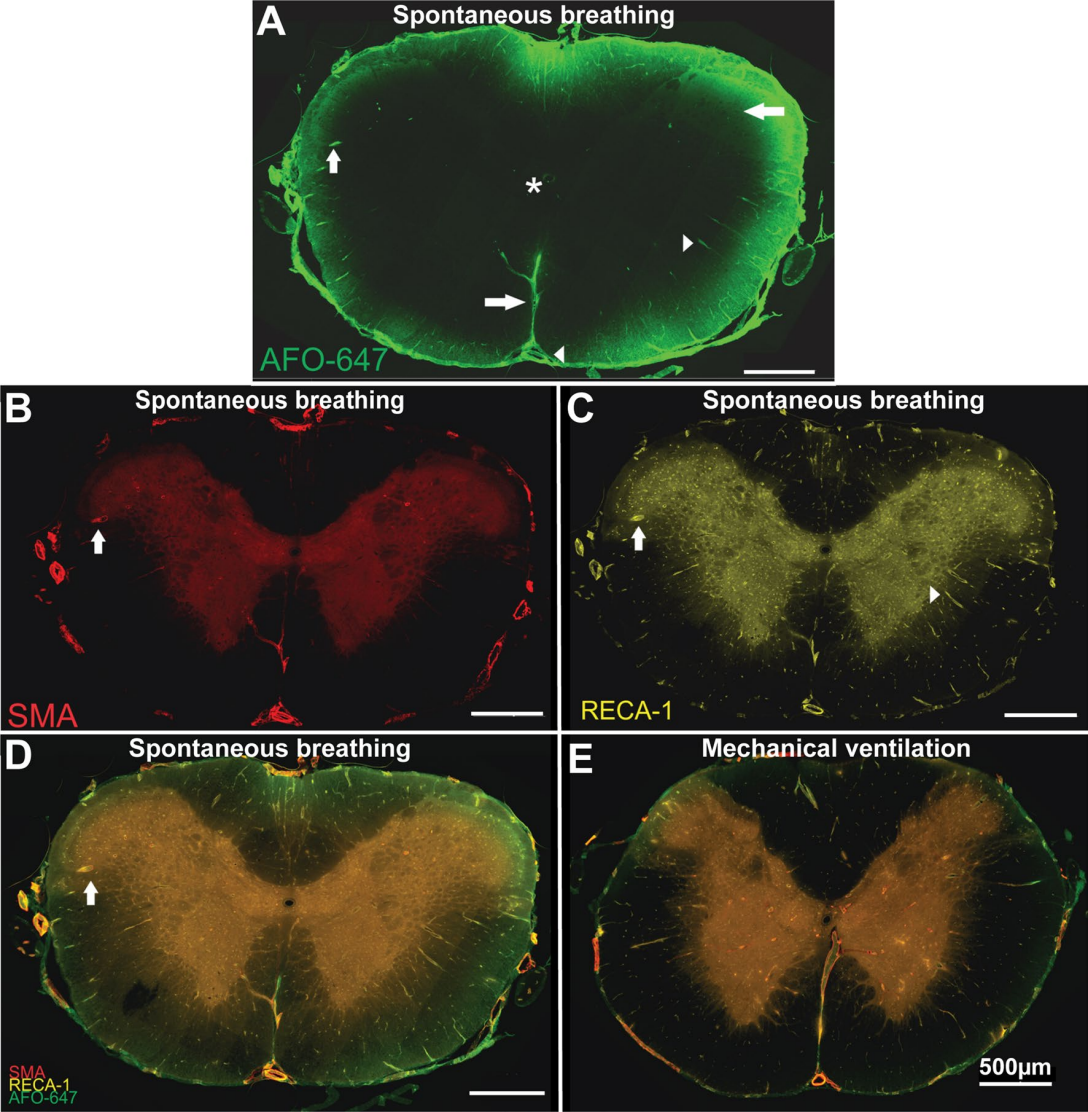
Tracer inflow into the spinal cord occurs via perivascular and transpial routes. **A** After injection into the cisterna magna in a spontaneous breathing rat, fluorescent ovalbumin (AFO-647) appeared to cross the pia circumferentially, diffusing into the spinal interstitium (left pointing arrow). Perivascular labelling was also prominent (right pointing arrowhead) with notable intramedullary penetration, towards the central canal (*). Note the deposition of AFO-647 tracer around the anterior spinal artery and the central branches distal to the ventral median sulcus (left arrowhead and right arrow respectively). **B**–**D** Tracer colocalised with both arterioles (up arrow) and venules (right arrowhead), confirmed with immunofluorescent stains for smooth muscle actin (**B**), and an endothelial cell marker (RECA-1) (**C**). The merged fluorescent channel is shown in **D**. In a mechanically ventilated control rat at C2 (**E**), there was reduced tracer signal across the whole axial section. Transpial fluorescence was conspicuously reduced, although perivascular deposition was still present

In all animals, AFO-647 deposited on the pial surface and around intramedullary and extramedullary blood vessels. A transpial, “parenchymal”, diffusive pattern of tracer transport was also observed (Fig. 6), but this was largely noted in spontaneous breathing rats. In this cohort, the transpial pattern was observed in 33% of spinal levels compared with just 10% in the *Normal* cohort, and none in the matched *Control* arm. In hypertensive and tachycardic rats, this transpial deposition of AFO-647 was found in 14% and 19% (respectively) of spinal levels, similar to that detected in the *Normal* group (Table 1). Note that tracer colocalisation to anatomical structures was noted as either present or absent (after ensuring uniform adjustment of brightness and contrast in every photomicrograph). Further quantitative or statistical analyses were not performed.

**Table 1:** Distribution of CSF tracer in the spinal cord

	Transpial %	White matter arterioles %	Grey matter arterioles %	Central canal %
Control	0	39	62	8
Normal	10	76	61	17
Spontaneous breathing	33	86	71	47
Hypertensive	14	62	33	19
Tachycardic	19	76	33	75

The percentage (%) of spinal levels with CSF tracer (AFO-647) deposited in the superficial spinal cord, adjacent to the pia (transpial), and around arterioles in the white and grey matter are shown for each cohort of animals, n = 6/7 rats

Labelling of large extramedullary vessels such as the anterior spinal artery was ubiquitous. In both the white and grey matter, tracer selectively accumulated around arterioles and venules, especially around radially projecting blood vessels (Fig. 6A–D). Pericapillary tracer was detected but was not as abundant as periarterial or perivenular deposition. In the positive pressure ventilated control groups, *Normal* and *Control*, tracer was identified around white matter arterioles in 76% and 39% of spinal levels respectively. In spontaneous breathing, hypertensive, and tachycardic rats this was observed in 86%, 62% and 76% of spinal levels, respectively. In the grey matter, periarteriolar tracer fluorescence was detected in 61% and 62% of spinal levels in *Normal* and *Control* cohorts, respectively. In spontaneously breathing animals, this phenomenon was observed more frequently (71%), while in both paced and hypertensive rats, this was observed less commonly (33% each).

**Fig. 7:**
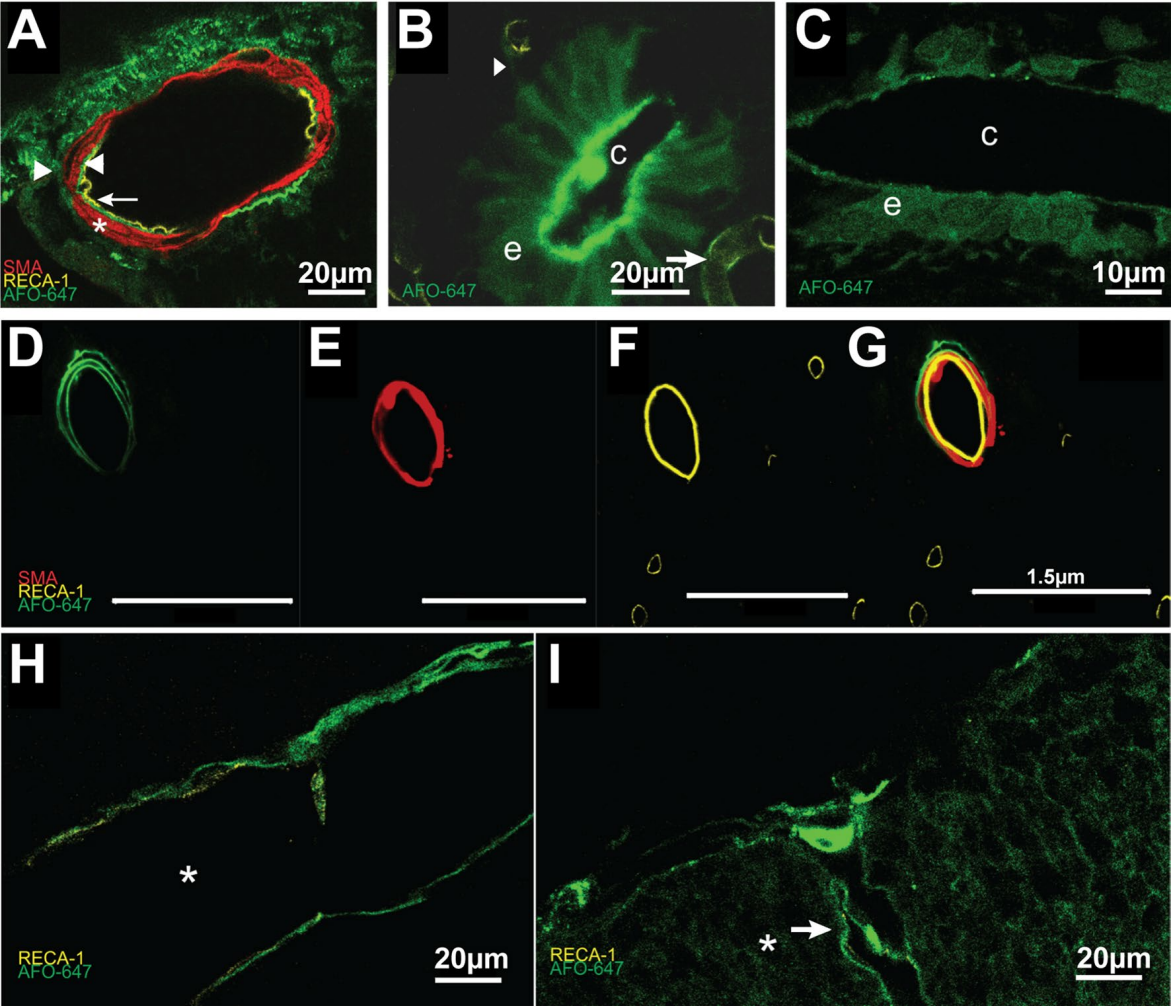
Tracer inflow occurs around venules, arterioles and the central canal. **A** An example of an anterior spinal artery under confocal microscopy. In all animals where extramedullary arterial fluorescent ovalbumin (AFO-647) deposition was evident, tracer was detected not only external to (right arrowhead) the smooth muscle layer (*) but also within, and internal to the tunica media (left arrow). Left pointing arrowhead denotes the endothelium. A 3D video of an anterior spinal artery (Additional file 7: Video S7) demonstrates this more clearly. **B**, **C** AFO-647 was detected within the ependymal layer of the central canal. There was an uninterrupted serpiginous trail of tracer (arrowhead) between subependymal microvessels and the central canal (right arrow marking venule, while c denotes the lumen of the central canal in **B** and **C**). This suggests privileged pathways may exist between the central canal and the subependymal perivascular spaces. AFO-647 tended to concentrate on the luminal aspect of the ependymal cells. Tracer was occasionally observed within the lumen of the central canal. Tracer unevenly deposited on the luminal side of the ependymal layer (note speckled appearance in **C**). The nuclei of ependymal cells were demarcated by tracer (marked by e), suggesting uptake by these cells. **D**–**G** Depicts multiple distinct layers of AFO-647 tracer within and external to the smooth muscle layer of an intramedullary grey matter arteriole (AFO-647, SMA, RECA-1 and merged channels are shown in **D**, **E**, **F** and **G** respectively). **H** AFO-647 accumulated around the outside of the endothelium of a large intramedullary venule (* marks the lumen). **I** A venule at the pial surface had similar deposition of AFO-647 around the endothelium (arrow). In **I**, * marks transpial migration of tracer, depositing in a convoluted, lattice pattern which reflects the spinal extracellular space architecture

In both extramedullary and intramedullary arteries and arterioles, confocal microscopy confirmed perivascular deposition of AFO-647 (Fig. 7A, D–G). Distinct layers of tracer were detected immediately external, within, as well as immediately internal to the smooth muscle cells of the tunica media of the anterior spinal artery. This was also observed in the arterioles of the vasocorona and within the grey matter. Tracer deposited immediately around the RECA-1 labelled endothelium of intramedullary venules and capillaries, as well as the extramedullary veins of the ventral median sulcus and the superficial pial network (Fig. 7H, I). Furthermore, at higher magnifications, deposition of AFO-647 in the spinal cord extracellular space highlighted the tortuous nature of the interstitial microarchitecture (Fig. 7I).

Tracer deposited around the central canal, but the spinal levels over which this was found were often non-contiguous. AFO-647 deposition around the central canal occurred in 47%, 17% and 8% of total spinal levels in spontaneous breathing, *Normal*, and *Control* cohorts, respectively. Hypertensive rats exhibited a similar incidence (19%), while tachycardic rats had increased tracer deposition around the central canal (75%) compared with the control groups. On confocal microscopy, tracer distributed heterogeneously within the ependymal layer of the central canal. There was preferential accumulation along the luminal border of ependymal cells, with less fluorescence along the abluminal aspect (Fig. 7C). On higher magnification, there was speckled clumping of tracer on the luminal aspect (Fig. 7C). It was not possible to confirm whether there was tracer uptake by ependymal cells. Occasionally, intraluminal tracer was detected. Serpiginous trails of tracer were also observed between the abluminal aspect of the ependymal layer and nearby subependymal microvessels (Fig. 7B). This suggested a pathway linking subependymal perivascular spaces and the central canal.

## Discussion

These data comprehensively establish that transport of CSF tracers in the spinal cord is dominated by respiratory-driven intrathoracic pressure changes, and that the cardiac pulsation component is substantially smaller. Moreover, these effects appear to play a major role in tracer influx into the spinal cord via perivascular spaces. Complementary in vivo and ex vivo imaging modalities characterised the effects of intrathoracic pressure and arterial pulsations on fluorescent tracers injected into the CSF. In the cervicothoracic subarachnoid space, alternating negative and positive intrathoracic pressures of spontaneous respiration were associated with greater CSF tracer redistribution compared with continuous positive pressures in mechanical ventilation. The same mechanisms likely exerted a dominant effect at a microscopic level, promoting tracer influx into the spinal interstitium via perivascular spaces and through the pial surface. Compared with respiration, arterial pulsations and tachycardia did not appear to play prominent roles in driving CSF tracer through the subarachnoid space or into the spinal interstitium. This is possibly related to arterial pulsations mediating influx through perivascular routes only, with minimal transpial transport.

### Transport of CSF tracers in the subarachnoid space

It has long been held that cardiac pulsations drive CSF flow in the cranial subarachnoid space [7, 24–27] and cardiac-gating imaging methods reinforced this belief. There is evidence from small historical investigations that respiratory pressures have a significant effect on spinal CSF [28]. A number of investigators posited that lumbar CSF pressure waves originate from spinal arterial pulsations with some contribution from the cranial vasculature (such as the choroid plexus) [29–31]. This is supported by at least one canine study involving spinal blocks and aortic occlusions [29]. Most of the data about physiological drivers of CSF flow have been derived from human MRI brain studies, and the role of respiration in spinal CSF dynamics is not clear. In these studies, the dural venous sinuses transmit pressure changes from the thorax and abdomen to the cranial subarachnoid space [32]. Recently, the Dreha-Kulaczewski group [33–35] demonstrated prominent rostral movements of CSF throughout the spinal canal at the beginning of forced inhalation in human subjects using real-time PC-MRI. Inspiration-induced CSF waves were more pronounced than those associated with cardiac pulsations, which were low amplitude. However, forced expiration resulted in caudal flow from T6 and very low flows rostral to T1–4. The authors hypothesised that a “watershed” point at the level of the heart divided the spinal subarachnoid space into two compartments. This in turn reflects the intimacy between intrathoracic and intraabdominal pressures, and the pressures within epidural venous plexus that are thought to modulate CSF dynamics. A recent study has emphasized this interaction between thoracic and abdominal pressure, reporting that it is the combination and balance of these pressures that govern spinal CSF movements [14].

The findings in our in vivo studies support previous MRI and computational experiments. We posit that the cyclical negative and positive pressure generated by the thoracic and abdominal cavities are transmitted directly to the continuous epidural venous–azygos venous system. The pliant thecal sac is, in turn, directly exposed to this venous network. Deformation of the dura drives CSF movement. Moreover, there is in silico evidence that pulsations in the subarachnoid space are transmitted to the microvasculature [7]. If negative intrathoracic pressures are eliminated, either increased positive pressure drives CSF cranially, or the movement of fluid even at the perivascular level is dampened. This was observed in our NIR and intravital experiments.

We described the first deployment of ICG and the IR800 NIR function on a commercial operating microscope to assess tracer movement in the spinal CSF in vivo in an animal model. This technique established that changes in intrathoracic pressures, not cardiac pulsations, affect the displacement and velocity of CSF redistribution. The intravital two-photon excitation microscopy demonstrated the dynamic nature of fluid movement in the spinal subarachnoid space. The ex vivo experiments confirmed that the dynamic flow of CSF observed in the subarachnoid space of spontaneously breathing animals correlated with quantitatively greater tracer penetration to the deep interstitium. Evidence from all three modalities taken together, suggest that the increased interstitial inflow of tracer is driven by greater displacement and mixing creating greater exchange of solute and fluid between the subarachnoid space and spinal cord, through the pial surface and at perivascular spaces.

In the current study, we attempted to individually manipulate respiration, blood pressure, and heart rate. However, there is increasing evidence that artificial ventilation exerts complex effects on cardiac pre- and afterload, analogous to the administration of vasopressors [36]. Moreover, central venous pressure may be sensitive to ventilatory parameters such as tidal volume [37]. Therefore, it is important to be cognizant that cardiorespiratory forces are inextricably linked, and it may be difficult to examine such physiological drivers in complete isolation. Future studies will need to consider central venous pressure measurements.

### Tracer influx into the spinal cord

Mestre et al. [15] employed two-photon intravital microscopy and particle tracking velocimetry on fluorescent microspheres injected into the cisterna magna of mice, to derive pial surface perivascular CSF flow velocities. By synchronising the electrocardiogram and respiratory waves to the velocity of the tracked microspheres, they demonstrated that the microsphere motion was yoked to the cardiovascular pulsations, respiration had less effect on CSF flow. They thus concluded that the displacement of the arterial walls acted as a “perivascular pump” driving CSF in the same direction as arterial blood flow. It has since been suggested that the arterial wall pulsations reported would be too small to drive net flow of CSF [38]. The authors then acutely raised the MAP of the mice with Angiotensin II and found that the particle velocity reduced, concluding that the increased arterial stiffness, induced by hypertension, diminished the efficacy of the “pump”. It should be noted however, that the authors did not evaluate the effect of cardiac-pulsation-related CSF changes in the subarachnoid space. Moreover, the blood vessels studied were not proven to be penetrating arterioles, but may have been pial arterioles, which likely confounds their results. Bedussi et al. [39] performed intravital confocal microscopy to track CSF infused microspheres on the surface of mouse brains. They also found that paravascular CSF flow is driven primarily by cardiac pulsations. They found no microspheres below the subarachnoid space, but unlike Mestre et al.’s findings, particles were present around both arteries and veins, albeit to a lesser degree. This group’s interpretation of the pulsatile nature of the bead movement was that arterial pulsations generated mixing in the penetrating vessel perivascular space. In a mathematical model, Bilston et al. [40] showed that a mismatch in relative timing in arrival of a CSF pressure wave in the spinal subarachnoid space and an arterial pulse could theoretically drive perivascular fluid inflow along a small artery entering at a right angle to the spinal cord. This has been subsequently shown to be a feasible mechanism in the spinal cord [41] but not the brain [42] due to differences in the cranial and spinal pressure pulse shapes.

**Fig. 8:**
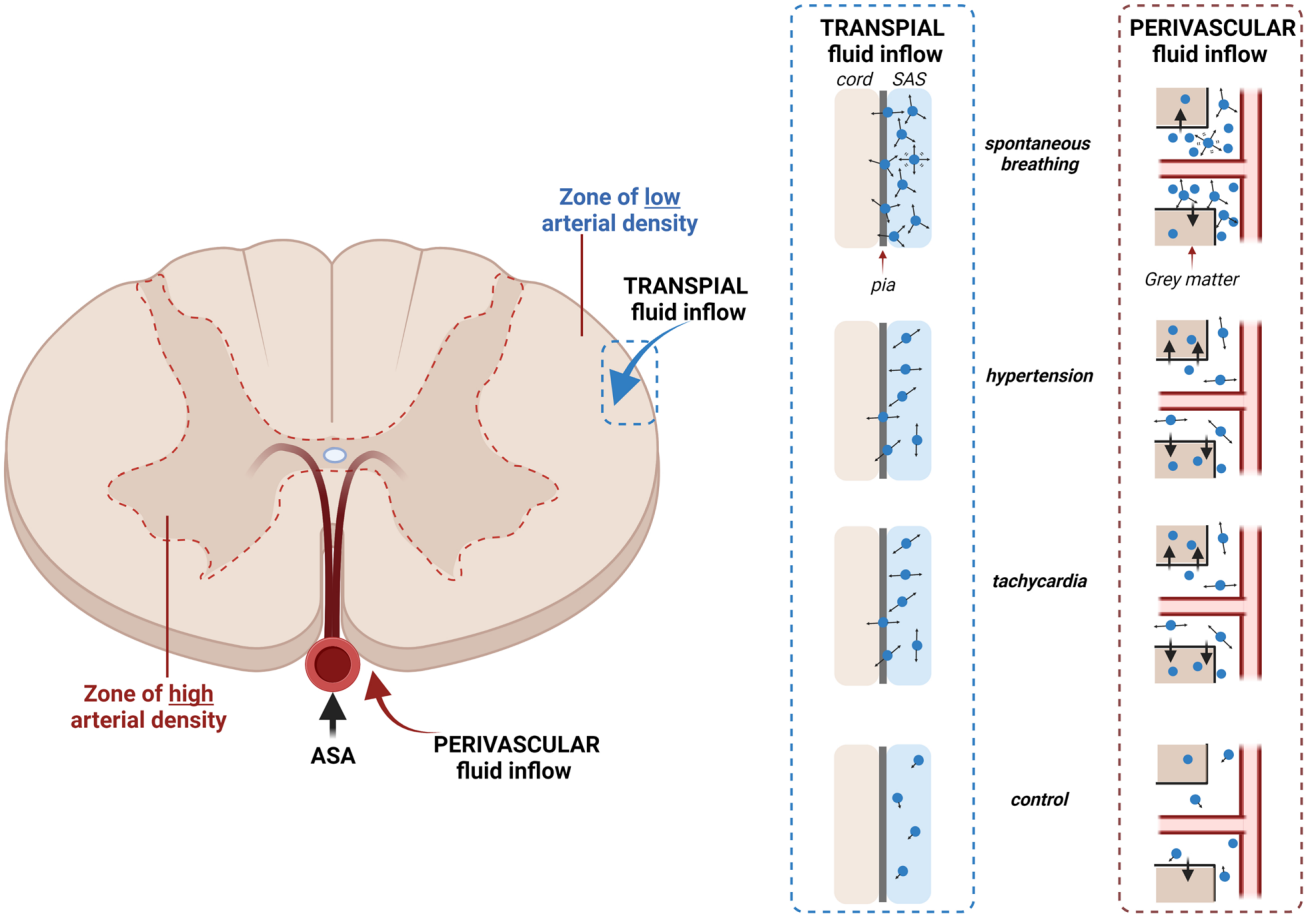
Schematic of spinal cord inflow. Respiration, specifically cyclical fluxes in intrathoracic pressure (spontaneous breathing) drive transpial fluid flow into the spinal white matter as well as perivascular fluid flow in the grey matter. Hypertension and tachycardia had little effect on transpial transport. It is possible that perivascular flow is also unchanged. However, there is increased tracer movement from perivascular spaces into the spinal interstitium, predominately in the grey matter. Arrow length and direction indicate displacement and velocity of solute/fluid particles. *ASA* anterior spinal artery, *SAS* subarachnoid space

Influx of tracer into the spinal interstitium involves both transpial as well as perivascular routes. Spontaneous breathing, but not hypertension and tachycardia, was strongly associated with transpial transport of tracer from the subarachnoid space into the spinal cord. Transpial migration could account for the significantly higher fluorescence signal in the white matter where there is a paucity of arterioles. In spontaneously breathing rats, microspheres in the CSF had larger amplitude oscillations and greater displacement (demonstrated in intravital studies, see Fig. 3, Additional file 3: Video S3, Additional file 4: Video S4, Additional file 5: Video S5, Additional file 6: Video S6). This supported the corresponding in vivo NIR studies that found that the tracer wavefront moved at a higher velocity in the cervical subarachnoid space. This increased displacement of tracers likely corresponds to greater mixing in the subarachnoid space and a greater ability to penetrate the pial boundary. The increase in overall fluorescence and “perivacular events” in the grey matter also suggests that the intrathoracic pressure fluxes that occur in spontaneous breathing are responsible for driving fluid along perivascular spaces, possibly via increased mixing on the surface of the spinal cord (Fig. 8).

In our intravital experiments, hypertension induced greater pulsatile “to and fro” movement but reduced net flow compared to spontaneously breathing animals. This correlated with the slower cranio-caudal movement of tracer in the spinal subarachnoid space with NIR imaging. A similar result was evident in tachycardic animals. The ex vivo studies indicated that this reduced mixing in the subarachnoid space translates to a reduction in transpial deposition of tracer (Fig. 4F). Interestingly, although in both hypertensive and tachycardic rats there was increased tracer fluorescence in the grey matter (Fig. 4G, J), this was not the result of an increase in “perivascular events” (Fig. 5E, G). It is possible that elevating the pulse rate and pressure does not result in an overall increase in perivascular tracer transport, but more tracer from the perivascular space is driven into the surrounding extracellular space. This would account for the increased fluorescence observed within the grey matter, as it is highly vascularized, supplied by the central branch of the anterior spinal artery. Indeed, tracer signal was relatively reduced within the white matter, which has comparatively fewer penetrating vessels. Although blood pressure and tachycardia appear to be involved in fluid transport, there is still inconsistent evidence from the literature and our experiments on the precise effects of hypertension and tachycardia on CNS fluid influx.

In ex vivo samples, AFO-647 selectively coalesced around blood vessels of all types. This confirmed the perivascular space as a privileged pathway for fluid and solute transport. Lam et al. [43] observed deposition of intracisternally injected AFO-647 tracer around spinal intramedullary arterioles, venules, and capillaries. Wei et al. [44] also observed preferential accumulation of cadaverine tracer around numerous blood vessels but did not clarify whether venules were present. Our findings do not support the proposed routes of fluid exchange in the popular glymphatics theory, wherein fluid and solute gain peri-arterial access to the CNS, flow through the extracellular space to collect around major draining veins and exit the interstitium via peri-venular pathways [17, 45, 46].

## Conclusions

These animal studies demonstrate that intrathoracic pressure changes associated with respiration drive CSF tracer movement in the spinal subarachnoid space and into the interstitium. Neither hypertension nor tachycardia alter subarachnoid space hydrodynamics substantially. Pulse pressure and rate likely drive tracer transport from perivascular spaces into the surrounding interstitium, but their overall impact is less than that of the respiratory cycle on net tracer influx into the spinal parenchyma. In pathologies where there is obstruction of the subarachnoid space, such as in Chiari malformation, arachnoiditis, or spinal deformity, macroscopic CSF flow can be altered and fluid exchange between the spinal cord and subarachnoid space may be impaired resulting in accumulation of fluid within the extracelluar space of the spinal cord. Understanding the precise normal mechanisms of fluid exchange between the subarachnoid space and CNS tissue may give insight into the pathophysiology of syringomyelia genesis and secondary spinal cord injury.

## Supplementary Information

**Additional file 1: Video S1.** In vivo imaging of spinal CSF flow in spontaneous breathing rats. Following extensive muscle dissection to expose the bony anatomy from occiput to T2, the CSF tracer indocyanine green (ICG) was infused into the cisterna magna. The intraoperative view under white light (*video on the right*) was captured and used to identify the injection site and each spinal level. A near infrared (NIR) filter on a commercial operating microscope was used to capture the corresponding fluorescent video simultaneously (*video on the left*).

**Additional file 2: Video S2.** In vivo imaging of spinal CSF flow in mechanically-ventilated rats. Following extensive muscle dissection to expose the bony anatomy from occiput to T2, the CSF tracer indocyanine green (ICG) was infused into the cisterna magna. The intraoperative view under white light (*video on the right*) was captured and used to identify the injection site and each spinal level. A near infrared (NIR) filter on a commercial operating microscope was used to capture the corresponding fluorescent video simultaneously (*video on the left*).

**Additional file 3: Video S3.** Movement of microspheres in the spinal subarachnoid space in a mechanically-ventilated control animal. Multi-photon intravital imaging was carried out to view the movement of intracisternally injected microspheres (*blue*). Fluorescent dextrans were injected intravascularly to visualise blood vessels. Individual microspheres were manually tracked. Tracked microspheres are labelled with crosses (+) and their tracks marked with dotted lines.

**Additional file 4: Video S4.** Movement of microspheres in the spinal subarachnoid space in a spontaneous breathing animal. Multi-photon intravital imaging was carried out to view the movement of intracisternally injected microspheres (*blue*). Fluorescent dextrans were injected intravascularly to visualise blood vessels. Individual microspheres were manually tracked. Tracked microspheres are labelled with crosses (+) and their tracks marked with dotted lines.

**Additional file 5: Video S5.** Movement of microspheres in the spinal subarachnoid space in a hypertensive animal. Multi-photon intravital imaging was carried out to view the movement of intracisternally injected microspheres (*blue*). Fluorescent dextrans were injected intravascularly to visualise blood vessels. Individual microspheres were manually tracked. Tracked microspheres are labelled with crosses (+) and their tracks marked with dotted lines.

**Additional file 6: Video S6.** Movement of microspheres in the spinal subarachnoid space in a tachycardic animal. Multi-photon intravital imaging was carried out to view the movement of intracisternally injected microspheres (*blue*). Fluorescent dextrans were injected intravascularly to visualise blood vessels. Individual microspheres were manually tracked. Tracked microspheres are labelled with white+ and their tracks marked with dotted lines.

**Additional file 7: Video S7.** A 3D rendering of an anterior spinal artery axial section. The CSF tracer AFO-647 (shown in *green*) was detected not only external to the smooth muscle layer (shown in *red*) but also within, and internal to the tunica media (shown in *yellow*).

## Data Availability

The datasets supporting the conclusions of this article are available from the corresponding author on reasonable request.

## Abbreviations

ANOVA: Analysis of variance
AFO-647: Ovalbumin Alexa-Fluor®-647 conjugate
ASA: Anterior spinal artery
bpm: Beats per minute
CNS: Central nervous system
CSF: Cerebrospinal fluid
DIVE: Deep in vivo explorer
HR: Heart rate
ICG: Indocyanine green
ISF: Interstitial fluid
MAP: Mean arterial pressure
MRI: Magnetic resonance imaging
NDS: Normal donkey serum
NIR: Near infrared
PC-MRI: Phase contrast magnetic resonance imaging
PFA: Paraformaldehyde
RECA-1: Rat endothelial cell antigen
SAS: Subarachnoid space
SB: Spontaneous breathing
SD: Standard deviation
SEM: Standard error of the mean
SMA: Smooth muscle actin
VBE: Vario beam expander
